# Awareness and practice of airway pressure release ventilation mode in acute respiratory distress syndrome patients among nurses in Saudi Arabia

**DOI:** 10.1186/s12912-024-01763-w

**Published:** 2024-01-30

**Authors:** Abdulelah M. Aldhahir, Abdullah A. Alqarni, Mohammed A. Madkhali, Hussain H. Madkhali, Abdullah A. Bakri, Mohammad A. Shawany, Ahmed H. Alasimi, Abdullah S. Alsulayyim, Jaber S. Alqahtani, Mohammed M. Alyami, Saeed M. Alghamdi, Omar A. Alqarni, Ali Hakamy

**Affiliations:** 1 Respiratory Therapy Department, Faculty of Applied Medical Sciences, Jazan University, Jazan, Saudi Arabia; 2 Department of Respiratory Therapy, Faculty of Medical Rehabilitation Sciences, King Abdulaziz University, Jeddah, Saudi Arabia; 3 Respiratory Therapy Unit, King Abdulaziz University Hospital, Jeddah, Saudi Arabia; 4 Department of Respiratory Therapy, Georgia State University, Atlanta, GA USA; 5 Department of Respiratory Care, Prince Sultan Military College of Health Sciences, Dammam, Saudi Arabia; 6 Respiratory Therapy Department, Batterjee Medical College, Khamis Mushait, Saudi Arabia; 7 Clinical Technology Department, Respiratory Care Program, Faculty of Applied Medical Sciences, Umm Al-Qura University, Makkah, Saudi Arabia

**Keywords:** ARDS, APRV, Nursing, Saudi Arabia, Mechanical ventilation

## Abstract

**Background:**

This study aimed to assess the knowledge and current practice of using the airway pressure release ventilation (APRV) mode with acute respiratory distress syndrome (ARDS) patients and identify barriers to not using this mode of ventilation among nurses who work in critical areas in Saudi Arabia.

**Methods:**

Between December 2022 and April 2023, a cross-sectional online survey was disseminated to nurses working in critical care areas in Saudi Arabia. The characteristics of the respondents were analyzed using descriptive statistics. Percentages and frequencies were used to report categorical variables.

**Results:**

Overall, 1,002 nurses responded to the online survey, of whom 592 (59.1%) were female. Only 248 (24.7%) nurses had ever used APRV mode, whereas only 229 (22.8%) received training on APRV mode. Moreover, 602 (60.0%) nurses did not know whether APRV was utilized in their hospital. Additionally, 658 (65.6%) nurses did not know whether APRV mode was managed using a standard protocol. Prone positioning was the highest recommended intervention by 444 (43.8%) when a conventional MV failed to improve oxygenation in patients with ARDS. 323 (32.2%) respondents stated that the P-high should be set equal to the plateau pressure on a conventional ventilator, while 400 (39.9%) said that the P-low should match PEEP from a conventional ventilator. Almost half of the respondents (446, 44.5%) stated that the T-high should be set between 4 and 6 s, while 415 (41.4%) said that the T-low should be set at 0.4 to 0.8 s. Over half of the nurses (540, 53.9%) thought that the maximum allowed tidal volume during the release phase should be 4–6 ml/kg. Moreover, 475 (47.4%) believed that the maximum allowed P-high setting should be 35 cm H_2_O. One-third of the responders (329, 32.8%) stated that when weaning patients with ARDS while in APRV mode, the P-high should be reduced gradually to reach a target of 10 cm H_2_O. However, 444 (44.3%) thought that the T-high should be gradually increased to reach a target of 10 s. Half of the responders (556, 55.5%) felt that the criteria to switch the patient to continuous positive airway pressure (CPAP) were for the patient to have an FiO2 ≤ 0.4, P-high ≤ 10 cm H_2_O, and T-high ≥ 10 s. Lack of training was the most common barrier to not using APRV by 615 (61.4%).

**Conclusion:**

The majority of nurses who work in critical care units have not received sufficient training in APRV mode. A significant discrepancy was observed regarding the clinical application and management of APRV parameters. Inadequate training was the most frequently reported barrier to the use of APRV in patients with ARDS.

**Supplementary Information:**

The online version contains supplementary material available at 10.1186/s12912-024-01763-w.

## Introduction

Airway pressure release ventilation (APRV) is a mechanical breathing mode that alternates between two degrees of continuous positive airway pressure (CPAP) support [1, 2]. It also allows for spontaneous respiratory effort at any CPAP intensity. It is seen as a potentially life-saving method for patients suffering from acute respiratory distress syndrome (ARDS) who are struggling to maintain oxygenation [3]. APRV is a safe and effective technique for breathing that is pressure-limited, time-triggered, and time-cycled [1, 2]. The APRV mode employs CPAP with an inverse ratio of inspiration to expiration time (I = E) and can facilitate unrestricted spontaneous breathing in all ventilator cycles to make patients comfortable [4]. APRV has been found to give lower peak pressure, better oxygenation, less circulatory loss, and better gas exchange than conventional ventilation, without worsening the ARDS patient’s hemodynamic status [5]. This method is thought to aid in the goal of recruiting consolidated lung regions and preventing recurrent opening and closing of the alveoli (decruitment) [3]. Research has demonstrated that, when compared to other ventilation modes in ARDS patients, the APRV mode increased gas exchange and arterial oxygenation (PaO_2_/FiO_2_) ratios [6, 7]. Previous literature has elucidated that the timely implementation of APRV in adult patients with ARDS is associated with improvements in oxygenation status, respiratory compliance, and a reduction in the duration of both mechanical ventilation and intensive care unit (ICU) stays [8, 9].

Despite its widespread utilization as an ARDS rescue therapy in many ICUs worldwide, the terminology and settings for the APRV mode may differ slightly, but the concepts remain similar to those of other traditional modes [2, 10]. APRV settings encompass four fundamental parameters: P-high, T-high, P-low, and T-low. The term P-high denotes the heightened continuous positive airway pressure (CPAP) level sustained for an extended duration (T-high), aiming to facilitate optimal lung volume and alveolar recruitment. Conversely, P-low signifies a brief application of low CPAP pressure during a short period (T-low), wherein the majority of ventilation processes occur [2, 11]. Established protocols for APRV recommend setting P-high equivalent to the plateau pressure (Pplat) observed during conventional mechanical ventilation (CMV), while maintaining P-low at 0 cm H_2_O to prevent alveolar erosion during the release phase. Furthermore, initiating T-high within the range of 4 to 6 s is advised to sustain optimal minute ventilation, with T-low adjusted to achieve the end of expiratory airflow at 75% of the peak expiratory flow rate (PEFR) [12, 13].

It is not widely known how the APRV mode is utilized in the management of ARDS patients by nurses who work in critical care units, and what barriers there are to using the APRV mode. As a result, we hypothesize that nurses lack proficient knowledge regarding the effective application of APRV to patients diagnosed with ARDS. Thus, the study aimed to assess APRV utilization by nurses who work at critical care units in the management of ARDS patients and identify the most common barriers to not using APRV mode.

## Method

### Study design and instrument

A cross-sectional design was used in this study. The survey was developed, formulated by experts who had experience using APRV mode (ICU physician, respiratory therapist and ICU nurse) and consisted of three parts, 24 questions. The first part was about demographic data, the second part involved knowledge and clinical practice of the APRV mode, the selection of this part from and the strategies were used in APRV mode were from previous literature [12–15]. The last part was about barriers of not using the APRV mode. The questionnaire was tested and evaluated by ten ICU nurses to ensure clarity and comprehensibility (Supplementary 3).

### Data collection and sampling

Data collection was conducted and assembled through the SurveyMonkey platform from December 2022 to April 2023. Invitations to nurses were sent via professional organizations established on social media platforms, as well as the Saudi Nurses Association and the Saudi Nursing Society. Nurses employed in critical units in the Kingdom of Saudi Arabia (KSA) were the main target population of this study. Before starting the questionnaire, information about the study as well as chief investigator contact information were available. Additionally, a written informed consent was obtained from each participant, and voluntary participation was ensured. Participants can only answer the survey link once and the expected time to complete the survey was 10 min.

### Data analysis

The data were analyzed using descriptive analysis. Frequency and percentages were used to summarize the results. Mean and standard deviation were used to calculate the number of ARDS patients cared for per shift. The Statistical Package for the Social Sciences (SPSS) version 29 was used to perform the statistical analysis.

### Ethical approval

Ethical approval for this study was sought from the bioethical committee at Jazan University with reference number (44/04/364). The study has been conducted in accordance with the Declaration of Helsinki.

## Results

### Demographic data of the study participants

Overall, 1,002 nurses who worked in critical units completed the online survey. More than half of the participants were female (592, 59.1%). The majority of participants were from the central region of Saudi Arabia (410, 40.9%), followed by the western region (303, 30.2%). Most of the participants had a bachelor’s degree (746, 74.5%). The largest group of participants worked at the Ministry of Health hospitals (372, 37.1%), and had one to five years of clinical experience. The mean (SD) ARDS patients cared for per shift was between 2 and 3 (Table 1). Only one quarter (248, 24.7%) of the nurses had ever used APRV mode, whereas only 229 (22.8%) had received training on APRV mode. Moreover, well over half (602, 60.0%) of the nurses did not know whether APRV was utilized in their hospital. Additionally, two-thirds (658, 65.6%) of the nurses did not know whether APRV mode was managed using the standard protocol with ARDS patients. The full demographic data of the study participants are shown in Table 1.

**Table 1:** Demographic characteristics of ICU nurses (*n* = 1,002)

Demographics	Values, n (%)
**Gender**	
Female	592 (59.1%)
Male	410 (40.9%)
**Regions**	
Central Region	410 (40.9%)
Western Region	303 (30.2%)
Southern Region	228 (22.8%)
Eastern Region	40 (4.0%)
Northern Region	21 (2.1%)
**Academic qualification**	
Associate degree	54 (5.4%)
Bachelor degree	746 (74.5%)
Master degree	103 (10.3%)
Doctorate degree	98 (9.8%)
**Place of work**	
Ministry of health hospitals	372 (37.1%)
Ministry of defense hospitals	222 (22.2%)
Ministry of national guard health affairs hospitals	190 (19.0%)
King Faisal specialist & research center	77 (7.7%)
Ministry of interior hospitals	56 (5.6%)
University hospitals	22 (2.2%)
Royal commission hospitals	16 (1.6%)
Private hospitals	13 (1.3%)
Others	34 (3.3)
**Years of clinical experience**	
Less than 1 year	112 (11.2%)
1–5 years	533 (53.2%)
6–10 years	274 (27.3%)
More than 10 years	83 (8.3%)
**Number of ARDS patients cared for per shift**	2 ± 3
**Used APRV**	
Yes	248 (24.7%)
No	754 (75.3%)
**Received training on APRV mode**	
Yes	229 (22.8%)
No	773 (77.2%)
**Utilization of APRV mode in your hospital**	
Yes	184 (18.4)
No	216 (21.6%)
I don’t know	602 (60.0%)
**Existing APRV protocol at my hospital**	
Yes	132 (13.2%)
No	212 (21.2%)
I don’t know	658 (65.6%)

Data are presented as frequency and percentage or mean and SD

*Abbreviations*: *ARDS* Acute respiratory distress syndrome, *APRV* Airway pressure release ventilation

### Indications and initial settings of APRV mode with ARDS patients

The majority of participants 672 (67.1%) recommended using APRV mode with ARDS patients, followed by COVID-19 patients 468 (46.7%), patients with pneumonia 362 (36.1%), asthma patients 176 (17.6%), patients with pulmonary edema 174 (17.4%), patients with obesity-induced hypoventilation syndrome 148 (14.8) and the lowest percentage was with sleep apnea patients 57 (5,7%). Additionally, 444 (43.8%) of the participants chose prone positioning as the next strategy to improve oxygenation in ARDS patients when conventional M.V fails to improve oxygenation (please see Table 2).

**Table 2:** Indications and practice of using APRV mode with ARDS patients (*n* = 1,002)

Variables	Values, n%
***Section 1****: indication and other strategy to improve oxygenation*	
**Conditions APRV mode utilization is indicated**	
ARDS	672 (67.1%)
COVID-19	468 (46.7%)
Pneumonia	362 (36.1%)
Asthma	176 (17.6%)
Pulmonary edema	174 (17.4%)
Obesity-induced hypoventilation syndrome	148 (14.8%)
COPD	147 (14.7%)
Sleep apnea syndrome	57 (5.7%)
**Strategy to improve oxygenation when conventional MV fails to improve oxygenation**	
Prone positioning	444 (43.8%)
High-frequency oscillatory ventilation (HFOV)	191 (19.1%)
Airway pressure release ventilation (APRV)	157 (15.7%)
Extracorporeal membrane oxygenation (ECMO)	111 (11.1%)
Inhaled nitric oxide	60 (6.0%)
Other pulmonary vasodilators	39 (3.9%)
***Section 2***: *initial settings and management*	
**Initial P-high setting**	
Equal to the plateau pressure on conventional ventilator	323 (32.2%)
At 25 cmH_2_O	227 (22.6%)
Equal to the mean airway pressure on conventional ventilator	211 (21.1%)
2—5 cmH_2_O above mean airway pressure on conventional ventilator	170 (17.0%)
To achieve tidal volume of 6 ml/kg/pbw (predicted body weight)	71 (7.1%)
**Initial P-low setting**	
Match to PEEP from conventional ventilator	400 (39.9%)
0 cmH_2_O	217 (21.7%)
2—5 cmH_2_O	237 (23.6%)
Variable depending upon oxygenation	148 (14.8%)
**Initial T-high setting**	
2–3 s	171 (17.1%)
4–6 s	446 (44.5%)
Per desired minute ventilation and respiratory rate	260 (25.9%)
Per inspiratory to expiratory (I:E) ratio	125 (12.5%)
**Initial T-low setting**	
Set time (i.e. 0.4–0.8 s)	415 (41.4%)
Per inspiratory to expiratory (I:E) ratio	265 (26.4%)
When expiratory flow equals 25 – 49% peak expiratory flow	246 (24.5%)
When expiratory flow equals 50–75% peak expiratory flow	76 (7.6%)
**Maximum allowed tidal volume**	
4–6 ml/kg	540 (53.9%)
7–8 ml/kg	210 (21.0%)
9–10 ml/kg	134 (13.4%)
More than 10 ml/kg	59 (5.9%)
No limit	59 (5.9%)
**Maximum allowed P-high**	
30 cmH_2_O	123 (12.3%)
35 cmH_2_O	475 (47.4%)
40 cmH_2_O	324 (32.3%)
No maximum	80 (8.0%)
**Pressure support utilization during spontaneous breathing**	
Yes	588 (58.7%)
No	414 (41.3%)
***Section 3***: *weaning and discontinuation*	
**Criteria use to wean P-high**	
Reduce P-high gradually in attempt to reach a target of 20 cmH_2_O	234 (23.3%)
Reduce P-high gradually in attempt to reach a target of 15 cmH_2_O	309 (30.8%)
Reduce P-high gradually in attempt to reach a target of 10 cmH_2_O	329 (32.8%)
Reduce P-high gradually in attempt to reach a target of 5 cmH_2_O	130 (13.0%)
**Criteria use to wean T-high**	
Increase T-high gradually in attempt to reach a target of 7 s	252 (25.1%)
Increase T-high gradually in attempt to reach a target of 10 s	444 (44.3%)
Increase T-high gradually in attempt to reach a target of 15 s	245 (24.4%)
Increase T-high gradually in attempt to reach a target of 20 s	61 (6.1%)
**Criteria used to switch ARDS patients to CPAP**	
FiO_2_ ≤ 40%	167 (16.7%)
T-high ≥ 10 s	148 (14.8%)
P-high ≤ 10cmH_2_O	131 (13.1%)
(FiO_2_ ≤ 40%, T-high ≥ 10 s and P-high ≤ 10cmH_2_O)	556 (55.5%)

Data are presented as frequency and percentage

*Abbreviations*: *ARDS* Acute respiratory distress syndrome, *APRV* Airway pressure release ventilation, *COVID-19* Coronavirus disease of 2019, *COPD* Chronic obstructive pulmonary disease, *PEEP* Positive end-expiratory pressure, *CPAP* Continuous positive airway pressure

When APRV mode was initiated, 323 (32.2%) of the nurses recommended that the initial P-high settings should be equal to the plateau pressure on a conventional ventilator, while 400 (39.9%) recommended the initial P-low settings should match PEEP from a conventional ventilator. Furthermore, 446 (44.5%) recommended that the initial T-high settings should be between 4 and 6 s, while 415 (41.4%) recommended the initial T-low settings should be a set time (between 0.4 and 0.8 s). Of the 1,002 participants, 588 (58.7%) used pressure support during spontaneous breathing (please see Table 2).

### APRV mode management with ARDS patients

The maximum allowed tidal volume during the release phase recommended by 540 (53.9%) of the participants was between 4 and 6 ml/kg, whereas 475 (47.4%) recommended the maximum allowed P-high during the release phase to be 35 cm H_2_O (please see Table 2).

**Fig. 1:**
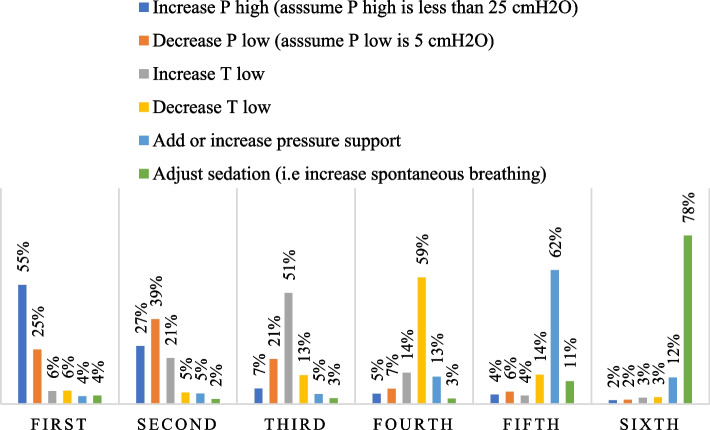
The order of interventions when levels of pH are unacceptably low and the PaCO_2_ is elevated in patients with ARDS (*n* = 1,002)

When using APRV in managing patients with ARDS, 553 (55.2%) of the nurses stated that increasing P-high (assuming P-high is less than 25 cm H_2_O), followed by 394 (39.3%) who stated that decreasing P-low, and 515 (51.4%) who stated that increasing T-low would be their first, second, and third prefatory choices to manage unacceptably low levels of pH with elevated partial pressure of arterial carbon dioxide (PaCO_2_) in patients with ARDS (please see Fig. 1 and Supplementary Table 1).

**Fig. 2:**
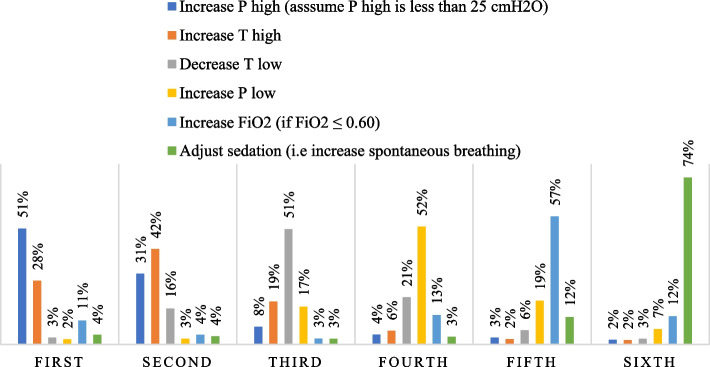
The order of interventions when levels of oxygen are unacceptably low in patients with ARDS (*n* = 1,002)

When using APRV in managing patients with ARDS, just over half of the nurses 515 (51.4%) chose increasing P-high (assuming P-high is less than 25 cm H_2_O), followed by 424 (42.3%) who preferred increasing T-high, and 513 (51.2%) who stated that decreasing T-low would be their first, second, and third prefatory choices to manage unacceptably low levels of oxygen in patients with ARDS (please see Fig. 2 and Supplementary Table 2).

### APRV Weaning and discontinuation in ARDS patients

One-third 329 (32.8%) of participants believed that when weaning ARDS patients, while in APRV mode, the P-high should be reduced gradually in an attempt to reach a target of 10 cm H_2_O, followed by 309 (30.8%) participants who thought that the P-high should be reduced gradually in an attempt to reach a target of 15 cm H_2_O. Moreover, 444 (44.3%) felt that the T-high should be gradually increased in an attempt to reach a target of 10 s, followed by 252 (25.1%) who thought that the T-high should be gradually increased in an attempt to reach a target of 7 s. When oxygenation goals are achieved and the ARDS patient is clinically stable, over half of the participants 556 (55.5%) thought that the criteria to switch the patient to CPAP would be to have an FiO_2_ ≤ 0.4, P-high ≤ 10 cm H_2_O, and T-high ≥ 10 s (please see Table 2).

### Common barriers to not using APRV mode with ARDS patients

**Fig. 3:**
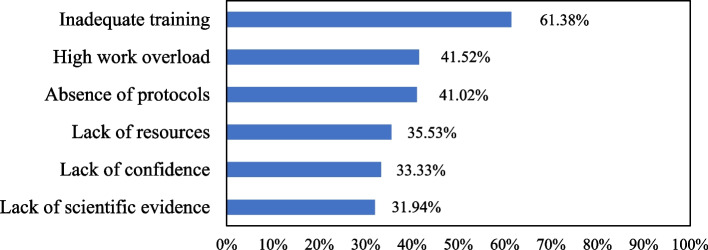
The most common barriers to using APRV mode (*n* = 1,002)

The most common barriers to not using APRV mode with ARDS patients from the perspective of nurses were inadequate training 615 (61.4%) followed by high work overload 416 (41.5%) and absence of protocols 411 (41.0%) (Fig. 3).

## Discussion

To the best of our knowledge, there is an extreme paucity of studies examining the clinical practice of APRV among nursing staff who work at critical units in Saudi Arabia. In light of this knowledge gap, this is the first national study to evaluate current awareness, practices, and barriers to the use of APRV mode in ARDS patients among nurses who work at critical units in the Kingdom of Saudi Arabia. Overall, our study’s results indicated that majority of nursing staff did not receive any training in utilizing APRV mode, and neither were they aware of APRV usage in their hospital nor the availability of APRV application guidelines in their facility. In addition, a significant discrepancy was found in nursing responses regarding indication, initial setting, weaning criteria, and discontinuation of APRV placement. However, nurses working in critical units revealed modest consistency in managing APRV settings to mitigate hypoxemia and hypercapnia.

Despite the enormous burden of the implementation of APRV in ARDS patients, APRV placement in the clinical setting remains controversial due to the lack of a standardized protocol [16, 17]. The clinical effectiveness of APRV mode has not been empirically proven in experimental studies due to the significant heterogeneity in APRV placement settings [18, 19]. Previous studies have indicated that RTs in Saudi Arabia were not well conversant in the application of APRV, where considerable disparities in setting and managing APRV parameters were observed as a result of a dearth of protocol and training seminars on the effective use of APRV [20, 21]. In line with this, our study outcomes found that nurses who work in critical units were not well-versed in APRV placement, as the vast majority (75%) had never used APRV mode, and a similar number (77%) had not received training in utilizing APRV mode. Furthermore, over half of the nurses (60%) were unaware of APRV usage in their hospital and two-thirds (66%) were not cognizant of the availability of APRV application guidelines at their facility. Thus, strict adherence to published guidelines, and receiving training in setting and adjusting the fundamental parameters of APRV, are indispensable prerequisites for obtaining the clinical benefit of the APRV application and avoiding life-threatening risks [12, 13].

Since the emergence of the APRV mode, it has contributed significantly as a rescue strategy for a wide spectrum of diseases, particularly ARDS [22]. Accordingly, the majority of the nurses in our study (67%) stated that ARDS was a primary indication for APRV application. Previous literature has attested to the clinical benefit of early APRV placement in adult patients with ARDS, as it substantially encourages spontaneous breathing, increases functional residual capacity (FRC), and thus optimizes ventilation/perfusion matching [2, 23]. A meta-analysis of randomized controlled trials (RCTs) has proven the significant advantages of APRV utilization, as it contributes to lower mortality, shortens the length of stay on a ventilator, and improves lung compliance and oxygenation status [24]. Notwithstanding, recent evidence has shown that the indication and effectiveness of APRV remain ambiguous due to a lack of robust evidence supporting the purported benefits of APRV [22, 25].

Understanding the complexity of APRV settings is vitally important for optimal clinical management [17]. Only one-third of the nurses (32%) reported that the P-high should match up with the plateau pressure (Pplat) on a conventional mechanical ventilator (CMV). These results are consistent with established APRV protocols that suggest setting P-high equal to the measured Pplat in volume control mode or similar to the set inspiratory pressure when switching from pressure control mode [12, 13]. A survey of 60 healthcare providers revealed that nearly half (48%) were strictly adherent to ARPV protocols for setting P-high [14]. Moreover, our study observed that approximately 40% of nurses indicated that P-low should be set to the same level as PEEP in a conventional ventilator. This result contradicts the APRV guidelines, which recommend initiating P-low at 0 cm H_2_O [12, 13]. These protocols were closely followed by healthcare professionals in the Miller et al. study, in which three-quarters (78%) set the initial P-value at 0 cm H_2_O [14]. It has previously been observed that setting P-low at 0 cm H_2_O generates an intrinsic PEEP that intentionally prevents alveolar erosion during the release phase because it will never permit expiratory flow to terminate below 25% of the peak expiratory flow rate (PEFR) [11, 26].

Furthermore, the results of our study indicated that almost half (45%) of the nurses thought that T-high should be started between 4 and 6 s, which matches the APRV protocols [12, 13]. Likewise, 65% of the participants in Miller et al.’s study showed identical findings in the T-high setting [14]. It is widely recommended that T-high not be set lower than 4 s to provide 8 to 12 releases per minute and consequently maintain optimal minute ventilation [2]. However, our study findings revealed that around 41% of nurses believed that the T-low should be set between 0.4 and 0.8 s. Similar disparities across healthcare practitioners were seen in Miller et al.’s study, with a significant number (39%) using an arbitrary T-low [14]. In contrast, several studies have shown the importance of adjusting the T-low to reach the end of expiratory airflow at 75% of the peak expiratory flow rate (PEFR) [27, 28]. In particular, appropriate adjustment of the T-low plays a pivotal role in stabilizing the alveoli by maintaining sufficient lung volume at the end of expiration to prevent periodic closure and reopening of the pulmonary units at low lung volumes [29, 30].

Regarding the management of the APRV settings, it was found that more than half of the nursing staff (54%) assumed that the maximum allowed tidal volume (V_t_) ranged between 4 and 6 ml/kg. In contrast, healthcare providers in the study by Miller et al. presented erratic results, with most of them (38%) stating that V_t_ should be set between 6 and 8 ml/kg [14]. Previous research has pointed out that tidal volume in APRV mode is controlled indirectly by adjusting T-low to maintain V_t_ between 4 and 6 ml/kg, thus optimizing alveolar ventilation [2]. Meanwhile, the expiratory time (T-low) should be effectively adjusted to be brief enough to prevent lung recruitment and long enough to attain an adequate tidal volume [11]. However, it was observed that nearly half (47%) of nurses believed that P-high should be limited to 35 cm H_2_O. In accordance with this, healthcare practitioners revealed similar findings in Miller et al.’s study, where 45% of them indicated the same limit [14]. It is strongly suggested to maintain a P-high less than 35 cm H_2_O and below the upper inflection point to reduce trans-alveolar pressure and hence decrease the risk of lung injury [2, 4, 31].

The results of our survey showed that increasing the P-high was the most dominant technique for enhancing ventilation and oxygenation status. It is well established that prolonged P-high is the first-line intervention in the management of respiratory acidosis and severe hypoxemia. Extended P-high can significantly promote alveolar recruitment by increasing mean airway pressure and lengthening gas exchange, resulting in better oxygenation levels and carbon dioxide clearance [11, 12]. Additionally, lowering the T-high and raising the T-low can be beneficial in improving alveolar ventilation because they provide greater time for exhalation and removal of PaCO_2_ [13]. Likewise, it has been shown that brief release periods at P-low can significantly improve pulmonary ventilation and lessen life-threatening hypercapnia [11, 26].

In terms of weaning APRV parameters, our study outcomes revealed that one-third (33%) of nurses believed that weaning of APRV mode should begin with a steady reduction in P-high to achieve 10 cm H_2_O, and 44% stated that a gradual increase in T-high should be applied to reach 10 s. These findings are in line with prior APRV protocols that advocated the “drop and stretch” approach to weaning APRV settings, which intended to decrease the P-high by 1 to 2 cm H_2_O and increase the T-high by 0.5 s for every 1 cm H_2_O drop in P-high [12, 13]. Furthermore, over half of nursing staff pointed out that an ARDS patient must meet certain criteria before converting to CPAP mode, including FiO_2_ ≤ 0.4, a P-high ≤ 10 cm H_2_O, and a T-high ≥ 10 s. Accordingly, subsequent studies have revealed the same criteria for switching an ARDS patient to CPAP mode with a PEEP value similar to P-high [11–13].

Although utilizing the APRV mode in patients with APRV has been associated with positive therapeutic outcomes, there are a variety of obstacles that may impede APRV implementation in the clinical setting. Our study analysis found that the most frequently mentioned barriers to adopting APRV mode are an absence of protocols, a heavy workload, and inadequate training. In agreement with this, it has been conclusively shown that the absence of randomized controlled trials is the primary impediment to the use of APRV in patients with ARDS [17, 18, 32]. A lack of consensus among practitioners in the initiation and management of APRV settings has been noted, which can be attributed to the absence of solid proof supporting the application of APRV placement [22, 33]. In light of the aforementioned findings, it is highly recommended to establish a training program and implement an institutional policy to improve nursing staff’s knowledge of the effective way to use APRV.

### Strengths and limitations

Our study is noteworthy because it is the first of its kind to evaluate Saudi Arabian nurses’ awareness, practice, and barriers related to using the APRV mode with ARDS patients. Additionally, it includes a sizable sample of nursing staff from different geographic areas, which facilitates the generalization of the results throughout the country. Nevertheless, certain limitations may hinder the scope of the research. This is a survey-based study that is unable to pinpoint the root cause of poor awareness of the APRV mode. In light of these limitations, further studies are warranted to examine the mechanism of action and to create evidence-based protocols for APRV mode in an attempt to raise the awareness of nursing staff regarding the optimal application of APRV.

## Conclusion

Overall, the majority of nurses who work in critical care units in Saudi Arabia did not receive sufficient training in APRV mode. A significant discrepancy was observed regarding the clinical application and management of APRV parameters. Inadequate training, a high workload, and a lack of guidelines were the frequently reported barriers to the use of APRV in patients with ARDS. A well-established evidence-based protocols and training programs for APRV mode are warranted to improve the clinical awareness of nursing staff regarding the optimal utilization of APRV.

### Supplementary Information

**Additional file 1.**

**Additional file 2: Supplementary Table 1.** The complete order for each intervention as reported by participants to manage unacceptably low levels of pH with elevated PaCO2 in patients with ARDS. **Supplementary Table 2.** The complete order for each intervention as reported by participants to manage unacceptably low levels of oxygen in patients with ARDS.

## Data Availability

The data used for the current study are available from the corresponding author on reasonable request.
