# Supplementary material for: Awareness and practice of airway pressure release ventilation mode in acute respiratory distress syndrome patients among nurses in Saudi Arabia
Source: BMC Nurs. 2024 Jan 30;23:79. doi: 10.1186/s12912-024-01763-w (PMC10826023; doi:10.1186/s12912-024-01763-w)
Supplement: Supplementary file 1 — Additional file 1. [file 12912_2024_1763_MOESM1_ESM.pdf]

Supplemntary 3:

Knowledge and current practice of using APRV in ARDS patients

Section 1: Demographic

1. Do you agree to participate in this study?

- Yes
- No

1. Gender
    - Male
    - Female

2. Geographical location
    - Eastern Region
    - Western Region
    - Central Region
    - Southern Region
    - Northern Region

3. Academic qualification:
    - Associate degree
    - Bachelor degree
    - Master degree
    - Doctorate degree

4. Place of Work:
    - Ministry of Health Hospitals
    - Ministry of National Guard Health Affairs Hospitals
    - Ministry of Defense Hospitals
    - Ministry of Interior Hospitals
    - King Faisal Specialist Hospitals & Research Centre (Riyadh, Jeddah, Medina)
    - Royal Commission Hospitals
    - University Hospitals
    - Private Hospitals
    - Other

5. Years of experience
    - Less than 1 year
    - 1-5 years
    - 6-10 years
    - More than 10 years

6. On average, how many ARDS patients are under your care per shift?

    Please sepecify___

7. Have you ever used APRV mode with ARDS patients before?
    - YES

- NO

8. Have you ever recived adequate training on APRV mode?
    - Yes
    - No

9. Is APRV used at your institution?
    - Yes
    - No
    - I don’t know

10. Is APRV mode managed via institutional protocol?
    - YES
    - NO
    - I don’t know

Section 2:Indications and initial settings of APRV

11. Which the following diseases/conditions are indications to use Airway pressure release ventilation (APRV) mode?
    - ARDS
    - COVID-19
    - Pneumonia
    - COPD
    - Pulmonary Embolism
    - Obesity
    - Asthma
    - OSA

12. When conventional mode of mechanical ventilation fails to improve oxygenation in ARDS patients, what is your next single strategy? (pick one)
    - APRV
    - HFOV
    - Prone positioning
    - ECMO
    - Inhaled nitric oxide
    - Other pulmonary vasodilator

13. What is the recommended initial P high setting with ARDS patients?
    - 25 cmH_2O
    - Equal to the plateau pressure on conventional ventilator
    - Equal to the mean airway pressure on conventional ventilator
    - 2-5 cmH2O above mean airway pressure on conventional ventilator
    - To achieve tidal volume of 6 ml/kg/pbw (predicted body weight)
    - Other

14. What is the recommended Initial P low setting with ARDS patients?
    - 0 cmH_2O

- 2-5 cmH2O
- Match to PEEP from conventional ventilator
- Variable depending upon oxygenation
- Others

15. What is the recommended initial T high setting with ARDS patients?
    - 2-3 seconds
    - 4-6 seconds
    - Per desired minute ventilation and respiratory rate
    - Per inspiratory to expiratory (I:E) ratio
    - others

16. What is the recommended initial setting for T low with ARDS patients?
    - Set time (i.e. 0.4 - 0.8 seconds)
    - Per desired inspiratory to expiratory (I:E) ratio
    - When expiratory flow equals 25-49% peak expiratory flow
    - When expiratory flow equals 50-75% peak expiratory flow
    - others

17. In ARDS patients, when the pH is unacceptably low and the PaCO2 is elevated, please rank your Adjustments by order? (ranking)
    A. Increase P high (assume P high is less than 25 cmH2O)
    B. Decrease P low (assume P low is 5 cmH2O)
    C. Increase T low
    D. Decrease T high
    E. Add or increase pressure support
    F. Adjust sedation (i.e. increase spontaneous breathing)
    G. Other (not specified)

18. In ARDS patients, when oxygenation is unacceptably low, please rank your Adjustments by order? (ranking)
    - Increase P high (assume P high is less than 25 cmH2O)
    - Increase T high,
    - Decrease T low
    - Increase P low
    - Increase FiO2 (if FiO2 $\leq$ 0.60)
    - Other (not specified)

19. During release phase, what is the maximum allowed tidal volume in ARDS patients?

    - 4-6 ml/kg
    - 7-8 ml/kg
    - 9-10 ml/kg
    - > 10 ml/kg
    - No limit

20. What is the maximum allowed setting for P high In ARDS patients?

    - 30 cmH2O
    - 35 cmH2O
    - 40 cmH2O
    - No maximum

21. Is pressure support used during spontaneous breaths with APRV in ARDS patients?
    - YES
    - NO

Section 3: APRV Weaning and discontinuation

22. In ARDS patients, which of the following criteria do you use to wean P high during APRV?
    - Reduce P high gradually in attempt to reach a target of 20 cm H_2O
    - Reduce P high gradually in attempt to reach a target of 15 cm H_2O
    - Reduce P high gradually in attempt to reach a target of 10 cm H_2O
    - Reduce P high gradually in attempt to reach a target of 5 cm H_2O

23. In ARDS patients, which of the following criteria do you use to wean T-high during APRV?
    - Increase T high gradually in attempt to reach a target of 7 seconds
    - Increase T high gradually in attempt to reach a target of 10 seconds
    - Increase T high gradually in attempt to reach a target of 15 seconds
    - Increase T high gradually in attempt to reach a target of 20 seconds

24. When oxygenation goals are achieved and the ARDS patient is clinically stable, which of the following criteria do you use to switch patient to CPAP?
    - $FiO_2 \leq 40\%$
    - P high ≤ 10 cm H_2O
    - T high ≥10 seconds
    - All criteria mentioned ($FiO_2 \leq 40\%$, Phigh ≤ 10 cm H_2O, and Thigh ≥10 seconds)
    - Others (Please specify) …………………………………………………………………….

Section 4: Barriers of not using APRV

25. What are the most common barriers of using APRV.
    - Inadequate training
    - Absence of protocols in my facility
    - High Work-load
    - Lack of confideince
    - Lack of resources
    - Lack of scientific evidence
