# Supplementary material for: Awareness and practice of airway pressure release ventilation mode in acute respiratory distress syndrome patients among nurses in Saudi Arabia
Source: BMC Nurs. 2024 Jan 30;23:79. doi: 10.1186/s12912-024-01763-w (PMC10826023; doi:10.1186/s12912-024-01763-w)
Supplement: Supplementary file 2 — Additional file 2: Supplementary Table 1. The complete order for each intervention as reported by participants to manage unacceptably low levels of pH with elevated PaCO2 in patients with ARDS. Supplementary Table 2. The complete order for each intervention as reported by participants to manage unacceptably low levels of oxygen in patients with ARDS. [file 12912_2024_1763_MOESM2_ESM.docx]

**Supplementary Table 1:** The complete order for each intervention as reported by participants to manage unacceptably low levels of pH with elevated PaCO_2_ in patients with ARDS.

| **Intervention** | **1^st^** | **2^nd^** | **3^rd^** | **4^th^** | **5^th^** | **6^th^** |
| --- | --- | --- | --- | --- | --- | --- |
| Increase P-high (assuming P-high is less than 25 cmH_2_O) | 553 (55.2%) | 269 (26.9%) | 72 (7.2%) | 47 (4.4%) | 44 (4.4%) | 17 (1.7%) |
| Decrease P-low (assuming P-low is 5 cmH_2_O) | 253 (25.3%) | 394 (39.3%) | 209 (20.9%) | 70 (7.0%) | 56 (5.6%) | 20 (2.0%) |
| Decrease T-high | 61 (6.1%) | 53 (5.3%) | 133 (13.3%) | 588 (58.7%) | 136 (13.6%) | 31 (3.1%) |
| Increase T-low | 60 (6.0%) | 214 (21.4%) | 515 (51.4%) | 145 (14.5%) | 39 (3.9%) | 29 (2.9%) |
| Adjust sedation (i.e. increase spontaneous breathing) | 39 (3.9%) | 23 (2.3%) | 27 (2.7%) | 25 (2.5%) | 106 (10.6%) | 782 (78.0%) |
| Add or increase pressure support | 36 (3.6%) | 49 (4.9%) | 46 (4.6%) | 127 (12.7%) | 621 (62.0%) | 123 (12.3%) |

**Supplementary Table 2:** The complete order for each intervention as reported by participants to manage unacceptably low levels of oxygen in patients with ARDS.

| **Intervention** | **1^st^** | **2^nd^** | **3^rd^** | **4^th^** | **5^th^** | **6^th^** |
| --- | --- | --- | --- | --- | --- | --- |
| Increase P-high (assuming P-high is less than 25 cmH_2_O) | 515 (51.4%) | 314 (31.3%) | 78 (7.8%) | 44 (4.4%) | 30 (3.0%) | 21 (2.1%) |
| Increase T-high | 283 (28.2%) | 424 (42.3%) | 191 (19.1%) | 60 (6.0%) | 24 (2.4%) | 20 (2.0%) |
| Increase FiO_2_ (if FiO_2_ < 0.60) | 107 (10.7%) | 43 (4.3%) | 27 (2.7%) | 131 (13.1%) | 569 (56.8%) | 125 (12.5%) |
| Adjust sedation (i.e. increase spontaneous breathing) | 43 (4.3%) | 36 (3.6%) | 25 (2.5%) | 34 (3.4%) | 122 (12.2%) | 742 (74.1%) |
| Decrease T-low | 31 (3.1%) | 160 (16.0%) | 513 (51.2%) | 210 (21.0%) | 63 (6.3%) | 25 (2.5%) |
| Increase P-low | 23 (2.3%) | 25 (2.5%) | 168 (16.8%) | 523 (52.2%) | 194 (19.4%) | 69 (6.9%) |
